# Self-management support needs for individuals with Myalgic Encephalomyelitis and their next of kin – a qualitative study

**DOI:** 10.1186/s12913-026-14962-9

**Published:** 2026-06-15

**Authors:** Kjersti Grønning, Liv Eli Lysfjord, Ann Katrin Hagen Røstad

**Affiliations:** 1 Department of Public Health and Nursing, Norwegian University of Science and Technology, Trondheim, 7491 Norway; 2 Nord-Trøndelag Hospital Trust, Postboks 333, Levanger, 7601 Norway

**Keywords:** Myalgic Encephalomyelitis, Chronic Fatigue Syndrome, Self-management support, Qualitative research, Patient-centred care, Digital health

## Abstract

**Background:**

Myalgic Encephalomyelitis/Chronic Fatigue Syndrome (ME/CFS) is a complex, disabling condition with limited evidence-based treatment options. Self-management support is recommended to improve people’s coping and quality of life, yet little is known about whether the provided self-management support meet individuals with ME/CFS and their next of kins needs. The aim of this study was to explore the self-management support needs of individuals with ME/CFS and their next of kin, and to identify barriers and facilitators to effective self-management support in order to inform improvements to existing self-management interventions.

**Methods:**

We conducted an exploratory descriptive qualitative study using a combination of semi-structured individual and focus group interviews with a total of 16 participants (12 individuals with ME/CFS and four next of kin) in Norway. Data were analysed thematically within a constructivist framework.

**Results:**

We identified three main themes. Theme one was named “Individualised and accessible support”, focusing on the importance of timing, readiness, and flexible delivery formats (digital, hybrid, modular). The second theme was named “Continuity and validation”, emphasising current gaps in follow-up care for individuals with ME/CFS and experiences of stigma. The third main theme was named “The role of peer support and practical strategies”, highlighting the value of peer interaction, sharing experiences, and adaptive tools (e.g., pacing, symptom tracking). Overall, the participants described that existing self‑management support was poorly aligned with their physical and cognitive limitations, lacked consistent and structured follow‑up, and often conveyed contradictory guidance on activity management.

**Conclusions:**

Self-management support for individuals with ME/CFS should be integrated into standardised care pathways, delivered in phased and modular formats, and include structured follow-up. Digital and hybrid solutions can enhance accessibility. Including peer-led components and family involvement may foster empowerment and reduce isolation. Training healthcare professionals in ME-sensitive communication and developing national guidelines are critical to improving service quality and reducing stigma.

**Supplementary Information:**

The online version contains supplementary material available at 10.1186/s12913-026-14962-9.

## Introduction

Myalgic encephalomyelitis (ME), also referred to as chronic fatigue syndrome (CFS), is a complex, multisystem condition characterised by persistent, abnormal fatigue accompanied by autonomic, neurological, and cognitive dysfunctions [1]. Estimates of the prevalence of ME/CFS vary considerably depending on the case definitions and diagnostic methods applied [2]. In the absence of recent large-scale, population-based studies assessing prevalence and incidence, the estimated prevalence in the United States is approximately 0.8% [3]. In Norway, a web-based survey of individuals with ME/CFS reported that 15% experienced a severe form of the condition, while 1% described their illness as very severe [4]. Despite its classification as a neurological disorder, ME remains poorly understood and under-recognised within many healthcare systems [5]. Evidence from a large study indicates that 97.8% of individuals reporting ME/CFS-like symptoms did not have a corresponding diagnosis recorded in their medical records, underscoring substantial underdiagnosis and limited recognition in clinical practice [6]. Delayed diagnosis is common and is associated with prolonged uncertainty for patients, as well as adverse physical and psychological consequences [7]. These challenges highlight the need to better understand the lived experiences of individuals with ME/CFS and to use this knowledge to inform the development of targeted interventions that support adaptive self-management over the course of the illness [8].

Despite being a serious and debilitating condition, evidence suggests that ME/CFS is not always recognised or taken seriously by healthcare professionals [9]. In response to growing concerns, the National Institute for Health and Care Excellence (NICE) published updated clinical guidelines in 2021 that explicitly emphasised the severity of ME/CFS [10]. The revised guidance advised against the use of graded exercise therapy and clarified that cognitive behavioural therapy should be offered solely as a supportive intervention to assist with symptom management and reduce distress, rather than as a curative treatment. This marked a substantial departure from the 2007 NICE guidelines [11] which had been widely criticised by patient organisations for their broad scope and for endorsing cognitive behavioural therapy and graded exercise therapy as evidence-based treatments for individuals with mild to moderate ME/CFS. Following sustained advocacy and pressure from patient groups, the guidelines underwent full revision through a process that was both highly scrutinised and contentious [1]. This evolution underscores the importance of developing treatment plans for ME/CFS through collaborative partnerships between patients and healthcare professionals, with an emphasis on shared decision-making and patient-centred care [1].

A recent systematic review and individual patient data meta-analysis examining the effects of cognitive behavioural therapy reported significant reductions in fatigue and functional impairment, alongside improvements in physical functioning among individuals with ME/CFS [12]. To date, the majority of research in this field has focused on biological mechanisms, with comparatively limited attention given to patient experiences and the organisation and delivery of health services [9]. Given the potentially profound impact of ME/CFS on individuals and their families, there is a clear need for research that explores self-management support needs among people with ME/CFS and their next of kin [5, 13, 14]. As there is no universally accepted “gold standard” definition of self-management [15], the present study conceptualises self-management needs as the requirements individuals have to manage symptoms, treatment demands, and the physical, psychosocial, and lifestyle consequences associated with living with a chronic condition.

To foster trust and improve quality of life among individuals with ME/CFS, digital health technologies have shown promising potential in supporting self-management in chronic conditions more broadly [16]. However, evidence regarding the effectiveness of digital health interventions specifically for supporting fatigue self-management among individuals with ME/CFS remains limited [14]. A Norwegian study involving professionals delivering digital self-management interventions found that such formats were perceived as offering fewer opportunities for interaction compared with traditional face-to-face approaches [17]. A recent review indicated that individuals with ME/CFS frequently use digital platforms to obtain information, share experiences, and seek emotional and social support [18]. Moreover, research suggests that digital health interventions may promote patient empowerment by facilitating active involvement in care, strengthening self-management capacities, and supporting sustained social participation [19, 20]. Given the central role of self-management in contemporary health policy, it is therefore important to explore the self-management support needs of individuals with ME/CFS and their next of kin [21] in order to strengthen knowledge on effective long-term approaches for this patient group.

On this background, this study aims to explore the self-management support needs among individuals with ME/CFS and their next of kin. Furthermore, the study seeks to identify barriers and facilitators to effective self-management support to inform enhancements to existing self-management interventions [5].

## Methods

An exploratory descriptive study design was employed [22, 23] to explore the self-management support needs of individuals with ME/CFS and their next of kin, and what this population considers important for the further development of self-management support services. The study was informed by a constructivist framework, which recognises knowledge as being co-constructed through ongoing interactions between the researcher, the participants, and the broader contextual setting [24].

The data collection was carried out through a combination of individual semi-structured interviews and focus group interviews [25]. This combination allowed for a comprehensive and complementary exploration of self-management support needs and facilitated the identification of barriers and facilitators to effective self-management support, informing the refinement of existing interventions. Individual interviews enabled in-depth exploration of participants’ personal experiences of living with ME/CFS and managing everyday challenges. This approach was particularly appropriate for exploring nuanced self-management needs, and perceived barriers that may be difficult to express in group settings. The interviews allowed participants to reflect on their illness trajectories, experiences with prior participation in hospital delivered self-management programs, thereby generating detailed, individual-level insights into self-management support experiences. In contrast, focus group interviews facilitated the exploration of shared and relational perspectives on self-management support in general. Participant interaction enabled the identification and elaboration of common experiences, collective challenges, and divergent views. Including both individuals with ME/CFS and their next of kin allowed studying how self-management support is negotiated within everyday relationships and caregiving contexts. Group discussions also illuminated facilitators and barriers related to service organisation, accessibility, and intervention delivery, as participants built on and responded to each other’s accounts. Together, these complementary methods enhance the richness, credibility, and comprehensiveness of the data, thereby strengthening the overall trustworthiness of the study [25].

### Study context

The study was conducted at the Department of Patient Education and Self‑Management Support at a local hospital in Mid‑Norway, serving a population of approximately 136,500 residents. The department offers a range of services and activities designed to support self‑management among individuals living with long‑term health conditions and their next of kin.

### Recruitment and data collection

#### Inclusion criteria

To capture self-management support needs among individuals with ME/CFS and their relatives, eligibility criteria were specified separately for the individual interviews and the focus group interviews. For the individual interviews, participants were required to have a diagnosed ME/CFS condition and to have participated in the group-based self-management course offered at the local hospital within the previous five years. For the focus group interviews, eligible participants included either individuals diagnosed with ME/CFS or relatives of individuals with ME/CFS who had experience with different interventions aimed at strengthening self-management in ME/CFS. Written informed consent was obtained from all participants prior to their participation in the interviews.

#### Recruitment

Participants were recruited using purposive sampling. For the individual interviews, 18 eligible individuals were invited, of whom four responded and provided written consent to participate. Interview times and locations were arranged on an individual basis to accommodate participants’ preferences and needs.

Recruitment for the focus group interviews was conducted in collaboration with the leader of the local ME Association, thereby incorporating patient and public involvement (PPI) into the study. The study was presented at a members’ meeting of the local ME Association, after which individuals diagnosed with ME/CFS and relatives of individuals with ME/CFS were invited to participate in the focus group interviews. In total, 12 individuals expressed interest in participation. The leader of the local ME Association worked in collaboration with the research team to organise the focus group sessions to accommodate participants’ preferences and needs.

#### Interview guides

The semi‑structured interview guides were informed by gaps identified in the existing literature on self‑management support needs among this population, as well as by clinical experience in delivering self‑management support to individuals living with a range of chronic conditions. The interview guides were developed collaboratively by the first (KG) and second (LEL) authors. They comprised open‑ended questions designed to elicit participants’ perspectives and experiences related to the study aims in their own words. These were supplemented with probing questions to promote elaboration and clarification, facilitate deeper reflection, and enable participants to expand on issues they considered important, thereby ensuring the generation of rich and nuanced data. The interview guides are provided as a supplementary file.

The individual interviews were conducted by the second author (LEL) in spring 2023, whereas the focus group interviews were jointly facilitated by the first and second authors (KG and LEL) in December 2024. The duration of the individual interviews ranged from 20 to 63 min, while the focus group interviews lasted between 82 and 85 min. Field notes were recorded during all interviews. All interviews were audio-recorded and transcribed verbatim by the two authors.

### Data analysis

The interview data were analysed using thematic analysis as outlined by Braun and Clarke [24]. This method is consistent with a constructivist epistemology, which recognises knowledge as co-constructed and acknowledges that data may be interpreted in multiple ways [26]. Thematic analysis offers an analytically flexible approach, with a explicit emphasis on researcher reflexivity throughout the analytic process [24, 27]. This approach enabled an in-depth exploration of participants’ self-management support needs and their perspectives on key considerations for the improvement of self-management services, ensuring attention to both content and modes of delivery. The initial phase of analysis involved familiarisation with the data, during which the authors (LEL and KG) transcribed all interviews verbatim and made reflective notes to identify salient statements and potential patterns. This was followed by systematic coding to identify recurring features in the data and organise them into initial codes [24]. Data extracts, including quotations, sentences, and longer passages, were coded inductively, resulting in the development of multiple codes. During this phase, data segments were not confined to a single code but could be assigned to multiple codes to capture the complexity of the material. In the subsequent phase, codes were examined and collated into broader themes in accordance with Braun and Clarke’s framework [24]. Related codes were grouped into overarching thematic categories, allowing patterns and relationships relevant to the research aim to be identified. At the conclusion of this stage, a preliminary thematic structure was developed, comprising three main themes and six sub-themes with associated codes, which formed the basis of the study’s findings.

The themes were then collaboratively reviewed and refined by both authors (LEL and KG) to ensure coherence, internal consistency, and alignment with the study aim. This process involved reorganising the thematic structure, merging certain themes, refining others, and clarifying boundaries between themes. Although the analysis was conducted in Norwegian, the final presentation of the findings was translated into English during the final phase of analysis.

### Ethical considerations

The study was conducted in accordance with the principles of the Declaration of Helsinki [28]. All participants were provided with both verbal and written information outlining the study’s purpose, emphasizing that their involvement was entirely voluntary. Written informed consent was obtained from all participants prior to the interviews. Once the recordings were transcribed and verified for accuracy, they were permanently deleted. To protect participant confidentiality, all personal identifiers were removed from the data, and no identifying information was linked to the transcripts. Prior to initiating the study, ethical approval was obtained from The Norwegian Regional Committees for Medical and Health Research Ethics (ref. 552188) and the Data Access Committee of the healthcare institution (ref. 2022/4776–36989/2022).

### Trustworthiness

The study was conducted in accordance with established criteria for trustworthiness in qualitative research, including credibility, transferability, dependability, and confirmability [29]. Credibility was strengthened through the researchers’ experience in patient education and self‑management support and by purposive recruitment of participants with relevant experience. As all authors were employed at the study site, reflexivity was maintained throughout to acknowledge researcher positionality and its potential influence on data collection and interpretation [24]. Reflexive discussions were conducted during data collection and analysis, including consideration of alternative interpretations and the influence of prior clinical and theoretical knowledge on coding and theme development. Preliminary findings were discussed with colleagues within the department to support analytic refinement. Transferability was supported through rich descriptions of the study context, participants, and analytic process, enabling readers to assess relevance to similar settings [30]. Authenticity was enhanced through the inclusion of illustrative participant quotations. Dependability was ensured by maintaining a consistent research team throughout data collection, transcription, and analysis, with close collaboration during coding and theme development to support consistency and transparency in the analytic process [30, 31].

## Findings

A total of 16 participants were included, consisting of 12 individuals diagnosed with ME (all female) and four next of kin (two male and two female), aged 22–75 years. All participants had over five years of experience with ME/CFS, either as patients or as next of kin.

Of the eight eligible individuals who met the inclusion criteria for individual interviews, four consented to participate. The reasons for declining participation in the individual interviews are unknown. In addition, 12 individuals (comprising both persons with ME/CFS and their next of kin) took part in the focus group interviews. Two focus groups were conducted, each consisting of six participants, with next of kin evenly distributed across the groups.

**Fig. 1:**
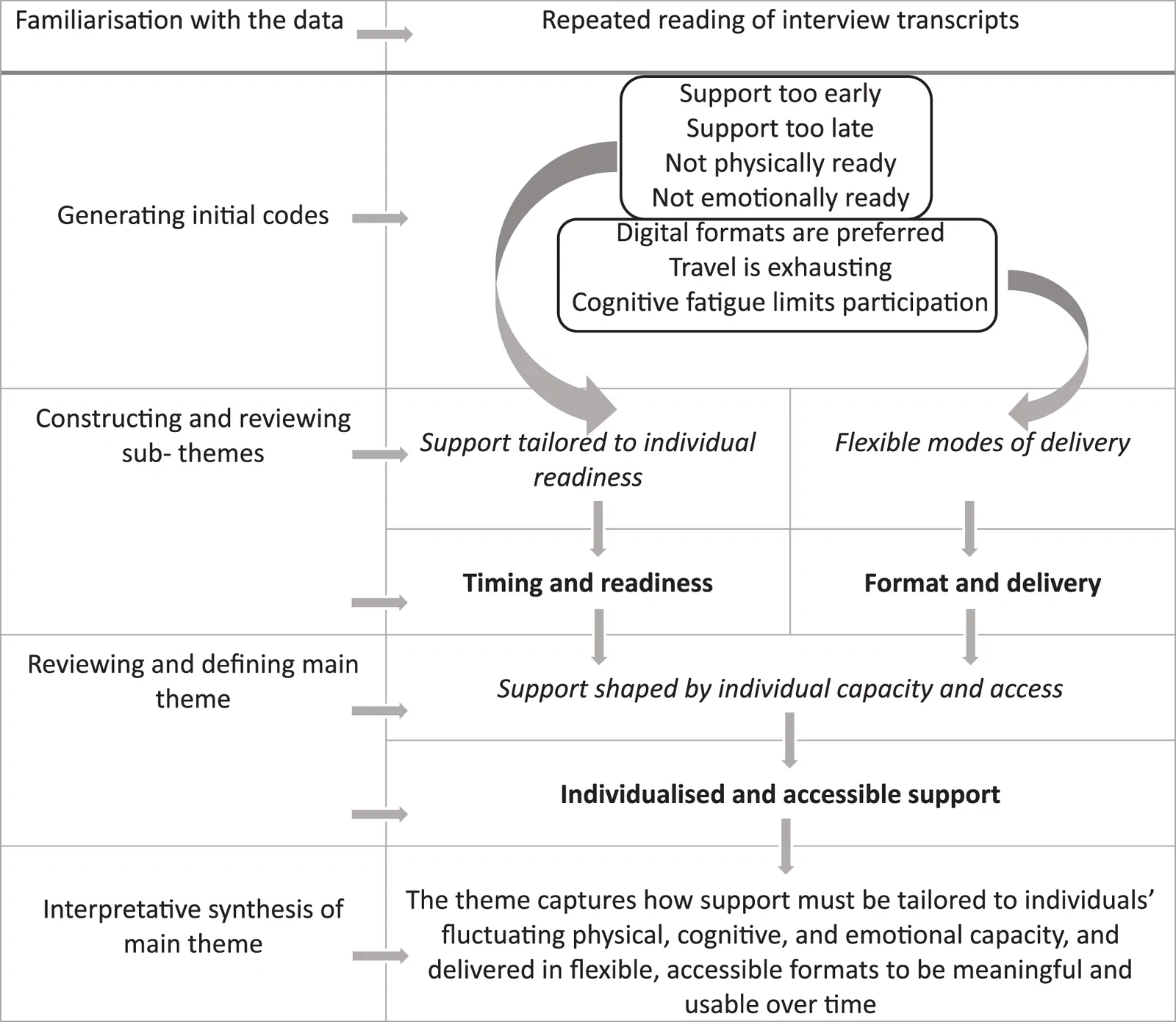
Illustration of the analytic process through which the theme *Individualised and accessible support* was developed. Final sub-themes and the main theme are presented in bolds

Through the reflexive thematic analysis, three overarching themes were constructed: (1) Individualised and accessible support (2), Continuity and validation, and (3) The role of peer support and practical strategies. Each theme was developed through iterative engagement with the data and further articulated through two related subthemes. Figure 1 presents an illustration of the analytic process through which theme *Individualised and accessible support was constructed*.

An overview of the constructed themes is presented in Table 1. To safeguard participant anonymity while demonstrating the breadth and variability of the dataset, quotations from the individual interviews and focus groups are labelled individual interview 1–4, and focus group 1–2, respectively.

**Table 1:** Overview of constructed themes

Main themes	Subthemes	Description
Individualised and accessible support	Timing and readiness	Support often arrives too early or too late, misaligned with patients’ physical and emotional readiness.
	Format and delivery	Need for flexible formats (digital, hybrid, modular) to accommodate energy levels, cognitive limitations, and geographic barriers.
Continuity and validation	Lack of follow-up	Patients feel abandoned after diagnosis or initial interventions, with no structured or ongoing support.
	Recognition and respect	Health professionals and society often misunderstand or dismiss ME, leading to stigma and emotional distress.
The role of peer support and practical strategies	Peer support	Shared experiences reduce isolation and foster emotional support, validation, and practical advice.
	Adaptive living tools	Patients rely on self-developed pacing strategies and seek structured guidance to manage daily life with ME.

### Individualised and accessible support

This theme captures the importance of tailoring the support to the unique and fluctuating needs of individuals with ME/CFS. The participants emphasised that timing, format, and delivery of support must be flexible and responsive to their physical and cognitive limitations, their life situation, and age.

#### Timing and readiness

Several participants described how the timing of current self-management courses or patient education interventions did not align with their personal readiness or life situation.

*I could have waited a bit longer. I was still in school and missed several classes….(Individual interview 3)*.

Others stated that they had received the self-management course when they needed, but this one-time offer was not enough.

*When you get the diagnosis*,* you don’t know anything. And then I got the course*,* which was really great…. However*,* it would have been nice to get a bit more follow-up afterwards. (Individual interview 1)*.

Some participants reported that self‑management support was offered at a point when they had not yet had sufficient time to emotionally process their diagnosis, while others described delays of several years before receiving any form of support. Such misalignment between the timing of support and individuals’ preparedness frequently resulted in disengagement or feelings of being overwhelmed.

The interviews also revealed that the participants readiness were not just about time since diagnosis, but also about emotional acceptance, symptom severity, and cognitive capacity. Several suggested that the self-management support should be offered in phases, and ideally in groups of people at similar stages of illness, to foster shared understanding and to reduce feelings of isolation.

*There are phases*,* we’re quite different. What works at one time doesn’t necessarily work at another. (Focus group interview 2).*

#### Format and delivery

Participants also emphasised the importance of accessibility and delivery format in relation to self‑management support. Travelling to attend in‑person courses was often described as overly demanding due to the constraints imposed by ME/CFS, particularly among those with severe symptoms. When invited to reflect on the potential of digital self‑management support as an alternative to travel, participants were generally positive. They discussed the advantages and limitations of various digital formats, including fully digital solutions, hybrid models combining digital and face‑to‑face elements, and modular resources that could be accessed on demand.

*Yes*,* I think so — maybe more people could have joined the course if it was digital and they wouldn’t have to show up in person. (Individual interview 2)*.

When reflecting on their experiences of in‑person, face‑to‑face self‑management courses, several participants reported that attendance often resulted in cognitive overload. This was primarily attributed to the simultaneous delivery of verbal information by healthcare professionals and the use of visual presentations, which participants found difficult to process concurrently. In addition, the overall course structure was perceived as insufficiently adapted to participants’ needs for frequent and shorter breaks. Participants consequently proposed several suggestions for improving the design of self‑management courses, including shorter sessions, increased opportunities for breaks, and simplified presentations tailored to the specific capacities and limitations associated with ME/CFS.

### Continuity and validation

This theme reflects participants’ expressed need for continuity of care and recognition from healthcare professionals. Many participants described the period of coming to terms with an ME diagnosis as emotionally challenging and marked by uncertainty. At the same time, receiving a formal diagnosis was often experienced as validating and relieving, as it represented acknowledgement of symptoms and challenges that participants felt had previously been questioned or dismissed.

*To accept that I actually have a diagnosis and start living accordingly is hard… Sometimes I’m not that bad*,* and other times I’m really unwell… (Individual interview 4)*.

*My doctor didn’t believe me — the general practitioner I had. (Focus group interview 1)*.

#### Lack of follow-up

Some participants described feeling abandoned following receipt of their diagnosis, reporting limited or no subsequent follow‑up from healthcare services. This lack of ongoing support was experienced as frustrating and contributed to a sense of being left to manage a complex and unpredictable illness independently, without sufficient guidance or regular updates

*About getting the diagnosis — the way I see it (as a next of kin)*,* the biggest problem is that it takes such a long time to understand what’s actually wrong with them. She was trying to work her way out of it. (Focus group interview 2)*

*I would have appreciated some follow-up afterwards. We’re often left on our own in different situations. (Individual interview 1)*

Participants further described a lack of continuity in care as particularly challenging, as it hindered their ability to manage the fluctuating nature of ME symptoms and the long‑term adjustments required to sustain quality of life. Attendance at a single self‑management course was widely perceived as insufficient on its own. Instead, participants expressed a need for more holistic and coordinated care, including ongoing self‑management support. They proposed individually tailored follow‑up, such as scheduled contacts at three‑, six‑, and twelve‑month intervals, to help maintain focus, reinforce key strategies, and foster a sense of continuity and connection. In addition, digital delivery formats, including video consultations and online group sessions, were suggested as more accessible alternatives for individuals with limited energy or mobility.

*But I’m thinking that… if there were something that wasn’t like traditional classroom teaching and instead included a small segment for social interaction — where you could meet others digitally — that would be helpful. You could access it when it suited you…….“I think that could be an advantage*,* allowing you to engage with it at your own pace.” (Focus group interview 1)*.

Participants further described how the lack of follow‑up limited their ability to remain informed about emerging research, treatment approaches, and coping strategies. In the absence of a clear point of contact or structured system for ongoing updates, many relied on self‑directed information seeking through social media or peer support groups, sources that were perceived to vary in quality and reliability. The absence of follow‑up was also linked to a broader sense of invisibility. Several participants described ME as a “lonely diagnosis”, noting that without regular contact or support from healthcare services, they often felt misunderstood, overlooked, and forgotten.

*You could use platforms like Zoom*,* where several participants can log in — even anonymously if needed — and speak to each other simultaneously. That might help reduce the workload for the course facilitator*,* especially when it comes to managing questions. Individual follow-up conversations could be offered afterwards. It’s just that… this is a lonely diagnosis. (Individual interview 2)*.

#### Recognition and respect

Recognition and respect constituted a salient theme of concern among the participants. Several had experienced that their symptoms were frequently dismissed, misinterpreted, or trivialised by healthcare professionals. Such invalidation not only amplified their psychological distress but also significantly undermined their confidence in the healthcare system.

*Being met with advice like ‘go for a walk’ or ‘start exercising’ when you’re that ill…. And then*,* at the very first appointment with the GP*,* we’re told: ‘You’re burned out. Here’s a prescription for antidepressants.’ You go home and say*,* ‘I’m not depressed. I don’t want to take this.’ And the response is*,* ‘Then don’t. It won’t make it worse or better.’ Giving medication for something you don’t have — couldn’t we instead take a moment to assess? To see whether it’s really depression*,* burnout*,* or possibly ME? (Focus group interview 2)*.

One participant also told that some instructors involved in self‑management support courses were perceived to have a limited understanding of ME. This lack of condition‑specific knowledge was described as affecting the relevance and perceived usefulness of the support provided.

*Unfortunately*,* some of the instructors in the self-management courses lack sufficient understanding. In fact*,* the lack of insight has been so noticeable that some participants have felt almost attacked — because ME is still not regarded as a ‘real’ diagnosis by some. There’s a significant lack of understanding within the healthcare system. I believe this is where we need to focus our efforts — to build awareness and understanding among healthcare professionals. (Focus group interview 2).*

Although some participants had negative experiences with the healthcare system, others talked about positive encounters and appreciated the professionals who listened attentively, validated their experiences, and tailored recommendations to their individual needs

*I have a fantastic GP — she’s great. She’s willing to try anything. (Focus group interview 1*)

### The role of peer support and practical strategies

This theme highlights how peer support and the sharing of practical strategies for managing everyday life with ME/CFS contributed to experiences of empowerment among the participants. Managing daily life with ME/CFS was described as a multidimensional and ongoing process involving the restoration of a sense of control, dignity, and personal agency in the context of a complex and often poorly understood condition. Several participants emphasised the role of peer support in facilitating a shift in perspective, from feelings of frustration and helplessness towards greater acceptance and self‑awareness. Sharing experiences related to practical self‑management strategies enabled participants to reframe their situation by focusing on their remaining capacities rather than perceived losses. For many, managing daily life with ME/CFS was not viewed as a fixed outcome, but as a gradual and evolving process shaped by individual insight, social connection, and continuous practical adaptation.

*I simply have to try to do something. It is about learning to cope with everyday life. Trying to engage in activities that provide motivation. I believe that a sense of mastery is extremely important. (Focus group interview 1).*

*Learning to shift thought patterns and reframe things — I found that to be very beneficial. (Individual interview 2).*

#### Peer support

Several participants identified the exchange of experiences within the group as the most valuable component of the self‑management support courses. Peer interaction was described as providing insights that were directly applicable to everyday challenges and as fostering a sense of mutual understanding and recognition. Some participants expressed a wish for greater emphasis on group discussions within self‑management courses, suggesting that such experiential exchange could complement, and in some cases be more valuable than traditional lecture‑based teaching formats.

*I believe peer support is incredibly valuable*,* as it helps people recognize themselves in others and feel less alone. However*,* there is far too little of it available. (Focus group interview 2).*

Participants highlighted the importance of connecting with others who shared similar experiences through structured group sessions, informal meetings, or digital communities. Such interactions were described as fostering a sense of belonging and validation, while also alleviating feelings of isolation commonly associated with living with ME/CFS.

*There are many possibilities. You could start with a larger group and then divide into smaller groups afterwards….“That way*,* you could say*,* ‘you’ll talk about this topic*,* and you’ll talk about that topic*,*’ and then regroup again afterwards. There are many ways to organize this digitally. I definitely think that could be a good solution — especially for those who live far away”. (Individual interview 4).*

Many participants emphasised that opportunities to exchange and listen to others’ lived experiences were perceived as more influential than formal instruction provided by healthcare professionals. They highlighted the value of peer‑based learning and expressed a need for greater integration of practical, individualised strategies within self‑management support courses. In addition, some participants described participation in various online peer groups, noting that informal conversations and sustained digital contact contributed to a sense of community and ongoing mutual support.

*At the time*,* I didn’t know much about autoimmune diseases. It’s something that doesn’t function properly and flares up unexpectedly. Over time*,* I’ve come to understand that rest is important. But back then*,* I didn’t receive much information — I had to seek it out myself. I joined Facebook groups for people with ME and talked to others who have the same diagnosis. That’s how you end up gathering knowledge on your own. You realize just how different we all are. (Focus group interview 2).*

#### Adaptive living tools

The sub‑theme adaptive living tools captures a range of strategies, aids, and adjustments described by participants as helpful in managing everyday life in accordance with fluctuating energy levels and physical limitations associated with ME. These tools extended beyond physical aids to include cognitive strategies, dietary adaptations, environmental modifications, and personalised routines. Collectively, such adaptations were described as reducing symptom burden and supporting more sustainable functioning in daily life.

*As mentioned*,* we are all on different paths*,* and while some react strongly to certain foods*,* others do not — but nutrition is extremely important. Even if you eat a healthy*,* traditional Norwegian diet and prepare everything from scratch*,* it may still not be entirely suitable. It needs to be individually tailored. If there are various recipes or dietary suggestions available that you can try out*,* that’s excellent. More knowledge is definitely needed. (Focus group interview 2).*

*They talked about increasing the amount of exercise every 14 days. That’s not something I can do. I have to take it day by day or hour by hour*,* really. One evening I might be able to stroll around the neighbourhood*,* and another day I can walk 6–7 kilometres. So*,* I can’t follow that kind of plan — I just go by how I feel that day. (Individual interview 1).*

The participants emphasised energy management as one key aspect of adaptive living. One participant described using a pulse watch to monitor exertion, planning rest periods after activity, and learning to recognise early signs of overexertion.

*And then there’s the pulse watch. I don’t exercise — I can’t even unload the dishwasher without my pulse shooting up. So*,* I monitor my body battery*,* stress levels*,* and pulse. Since I started using the watch*,* I actually feel it has helped a bit. (Focus group interview 2).*

Participants further described environmental adaptations as playing an important role in supporting everyday functioning. Several reported using measures such as noise‑cancelling earplugs or deliberately avoiding environments characterised by high levels of sensory stimulation, including loud noise or bright lighting. Others described adjustments to work arrangements, such as shorter working hours or delayed start times, to accommodate pronounced morning fatigue. These adaptations were often implemented through a combination of collaboration with accommodating employers and ongoing processes of trial and adjustment.

*You planned to visit a friend this afternoon — but it didn’t happen. It’s difficult*,* because I’ve kind of chosen work. That is because I think it’s so important to be employed. But maybe I’m making a mistake*,* you know*,* in terms of how my life is going — if that makes sense? (*Individual interview 4)

Cognitive and psychological strategies were also described as important adaptive tools. Participants referred to a range of techniques they had learned over time that supported coping with ME, including approaches to managing uncertainty, emotional strain, and fluctuating symptoms. These strategies were perceived as contributing to greater self‑awareness and resilience in everyday life.

*Yes*,* a kind of cognitive therapy*,* like changing my way of thinking a bit — instead of talking myself down. Because the body remembers what it used to be able to do*,* it holds on to that and wants to keep doing things the way it used to… So*,* I just started trying to accept things more… (individual interview 2).*

## Discussion

### Individualised and accessible support

This study explored the self‑management support needs of individuals with ME/CFS and their next of kin, with the aim of identifying barriers and facilitators that could inform improvements to existing interventions addressing the social and emotional challenges associated with living with a chronic and fluctuating condition [5]. Participants described self‑management as a dynamic and ongoing process involving strategies such as energy budgeting, pacing, symptom monitoring, and boundary setting. However, they emphasised that the perceived value and effectiveness of self‑management support were highly contingent on how, when, where, and by whom such support was delivered. These findings are consistent with existing evidence that conceptualises ME/CFS as a biologically grounded, multisystem disease frequently associated with cognitive impairment, thereby necessitating tailored and flexible support rather than standardised approaches based on uniform activity escalation [6, 9, 32].

A key barrier identified by participants was cognitive overload during intensive course sessions that combined visual presentations with concurrent verbal instruction. Such formats were reported to exceed participants’ capacity for sustained concentration and information retention. Cognitive dysfunction and limited benefit from traditional didactic education in ME/CFS have been well documented in previous research [9, 32]. To address these challenges, participants recommended the use of single‑channel content delivery, shorter sessions with regular breaks, and written materials to support recall and practical application. In addition, physical and logistical barriers, including severe fatigue, reduced mobility, and unfavourable sensory environments (e.g. noise and bright lighting), were described as further limiting access to in‑person support. Consistent with prior findings, these results underscore the importance of flexible self‑management support models, such as home‑based or digital delivery, shorter sessions, and sensory‑adapted environments [13, 19]. Digital solutions developed through participatory approaches have been shown to enhance patient empowerment, reduce travel‑related barriers, and facilitate continued peer interaction [20, 33]. Participants in the present study similarly emphasised that digital self‑management support should be interactive and modular, or available on demand, enabling engagement at times when energy levels permit, in line with findings from earlier studies [13, 17].

### Continuity and consistency

This study indicates that timing and continuity represent substantial challenges in the provision of self‑management support for individuals with ME/CFS. Support was frequently reported as being offered either too late, following prolonged diagnostic delays, or too early, at a point when individuals were too unwell to engage, and was rarely accompanied by structured follow‑up. To address these mismatches, embedding self‑management support within standardised care pathways may be beneficial [34, 35]. Integrating pacing guidance, individualised action plans, symptom monitoring tools, and health coaching within defined entry points, severity‑based stratification, and scheduled review appointments may facilitate the delivery of support at a point when individuals are clinically ready, rather than prematurely or belatedly. This approach is consistent with NICE guidance, which emphasises iterative, clinician‑supported energy management and ongoing follow‑up [10]. Furthermore, recent evidence suggests that clinical attention to post‑exertional malaise during consultations is associated with higher patient‑reported satisfaction and perceived benefit, underscoring the importance of structured pacing support and continuity of care [36]. The implementation of structured care pathways may also contribute to reducing diagnostic delays, which are common in ME/CFS and have been associated with poorer outcomes [7]. More broadly, existing research highlights the value of phased, well‑timed interventions combined with scheduled review points to support sustained behavioural adaptation in conditions characterised by symptom fluctuation [5, 21].

The findings also indicate that inconsistent and, at times, contradictory clinical advice such as recommendations for graded exercise alongside pacing, combined with dismissive attitudes, undermined trust in healthcare services. These experiences reflect ongoing debates surrounding psychosocial interventions, including variability in the effectiveness of cognitive behavioural therapy, and recent guideline shifts away from graded exercise therapy towards activity management tailored to post‑exertional malaise [1, 12]. Enhancing professional knowledge and ensuring consistent, post‑exertional malaise‑aware communication across services are therefore critical to preventing harm and strengthening therapeutic relationships.

### The role of peer support and practical strategies

Despite the challenges identified, participants consistently described peer interaction and the sharing of lived experiences as highly valuable in coping with ME/CFS. Meeting others facing similar challenges was repeatedly characterised as particularly meaningful, as it reduced feelings of isolation and facilitated the exchange of practical strategies for managing daily life. This finding is consistent with existing literature demonstrating that peer support can enhance emotional well-being and provide validation for individuals living with chronic conditions [14, 18]. Both online and group-based formats have been identified as important arenas for connection, particularly for individuals for whom physical attendance is limited by illness severity [13, 14, 17]. Integrating structured peer support, delivered either face to face or digitally, may therefore help address social and emotional needs alongside biomedical management [13, 19].

The study further showed that individuals with ME/CFS valued learning concrete and practical strategies, such as pacing, pulse monitoring, and energy budgeting, particularly when guidance was tailored to individual symptom patterns and variability rather than delivered as generalised activity recommendations. This finding aligns with previous research emphasising the importance of personalised approaches to self-management in ME/CFS [12, 13, 32]. Participants also highlighted the value of involving family members and other caregivers in self-management support, as this facilitated shared understanding, reduced household conflict, and promoted coordinated coping strategies aligned with patients’ needs [8, 32]. In addition, participants appreciated a strengths-based approach that focused on remaining capacities rather than limitations. Cognitive strategies promoting acceptance and self-compassion were perceived as particularly helpful, as they alleviated feelings of guilt and frustration, supported adjustment to fluctuating symptoms, and encouraged sustained engagement in self-management practices [12, 32].

### Strengths and limitations

A key strength of this study is its exploratory descriptive design combined with reflexive thematic analysis, which enabled in‑depth and nuanced exploration of the experiences of individuals with ME/CFS and their next of kin. The use of both individual and focus group interviews further strengthened the study by allowing insight into personal, sensitive experiences as well as shared and relational perspectives [25]. Individual interviews facilitated depth and reflection, while focus group discussions supported the exploration of collective experiences, contrasting views, and social dynamics, including relational aspects of illness and care through the inclusion of next of kin.

However, combining interview formats also introduced methodological challenges. Differences in interactional context may have influenced the type and depth of data generated, as some topics may be less readily expressed in group settings due to social desirability or confidentiality concerns. Integrating data from two formats required careful reflexive analysis and increased the overall research burden, which may be particularly demanding for individuals with chronic illness.

The study also has limitations related to sample composition. All participants with ME/CFS were women, which may have shaped the perspectives represented and limits the transferability of findings to other gender groups. In addition, participants were recruited from a single geographic area and were primarily individuals who had previously engaged in self‑management courses, potentially excluding those with more severe illness or limited access to services. Interviewing patients and next of kin together may have influenced participants’ accounts through relational dynamics; however, consistent with a reflexive thematic approach [24], these dynamics were considered part of the meaning‑making context rather than sources of bias. Finally, the analysis was conducted in Norwegian and subsequently translated into English, which may have introduced minor nuances in meaning despite careful verification. As with all qualitative research, the findings are context‑specific and not intended to be generalisable.

### Clinical implications

To strengthen self‑management support interventions for individuals with ME/CFS, several practical considerations should be prioritised. Early, pathway‑integrated support delivered shortly after diagnosis, together with structured and sustained follow‑up, may help ensure timely, appropriate, and clinically responsive care [7, 21, 35]. Group composition should be tailored according to factors such as illness severity, age, and employment status to enhance relevance and support meaningful peer interaction. Reducing cognitive load through adapted content delivery may accommodate difficulties with concentration and memory, while flexible delivery formats—including digital and hybrid approaches—can enable participation at times when energy levels permit. In addition, the integration of structured peer support, whether delivered face to face or online, may reduce social isolation and facilitate the exchange of practical self‑management strategies [14, 18].

### Future research recommendations

Future research should evaluate the effectiveness of phased and modular self‑management support interventions tailored to illness severity and patient readiness, using mixed‑methods or longitudinal study designs to capture changes over time. Further investigation is also warranted into digital and hybrid delivery models, with particular attention to their impact on accessibility, cognitive load, and patient engagement, especially among individuals with severe ME/CFS who are frequently excluded from in‑person programmes. Comparative studies examining peer‑led versus professionally facilitated self‑management support may help to elucidate the contribution of social support to empowerment and sustained engagement. In addition, research exploring family‑inclusive interventions and their effects on household dynamics and patient well‑being is needed. Finally, implementation studies focusing on the integration of self‑management support within standardised care pathways, including evaluations of cost‑effectiveness, scalability, and implications for health equity, will be essential to inform future policy and clinical practice.

## Conclusion

This study highlights a pressing need to strengthen self‑management support tailored to the specific challenges faced by individuals with ME/CFS and their families. Effective support should move beyond generic educational approaches and instead offer phased, individualised interventions that account for patients’ readiness, cognitive limitations, and fluctuating symptom patterns. Flexible delivery models, including digital and hybrid formats, modular content, and structured follow‑up, are essential to enhance accessibility and continuity of care. The integration of peer support and practical self‑management strategies, such as pacing and adaptive living tools, appears critical for fostering empowerment and reducing social isolation. In addition, targeted training for healthcare professionals is needed to promote ME‑sensitive communication, address stigma, and support respectful, collaborative care. Embedding self‑management support within standardised care pathways and developing national guidance may help address current service gaps, promote equity, and improve quality of life for people living with ME/CFS.

## Supplementary Information

Below is the link to the electronic supplementary material.

Supplementary Material 1

## Data Availability

The datasets used and/or analysed during the current study are available from the corresponding author on reasonable request.
