# Supplementary material for: Self-management support needs for individuals with Myalgic Encephalomyelitis and their next of kin – a qualitative study
Source: BMC Health Serv Res. 2026 Jun 15;26:1182. doi: 10.1186/s12913-026-14962-9 (PMC13508265; doi:10.1186/s12913-026-14962-9)
Supplement: Supplementary file 1 [file 12913_2026_14962_MOESM1_ESM.pdf]

Individual interview guide

- How long has it been since you received the ME diagnosis?
- How long after receiving the diagnosis did you participate in the self-management program?
- Was participating in the self-management program appropriate in relation to the time you received the diagnosis?
- Before attending the program, what did you know about ME and how to improve your everyday life?
    - Where did you seek knowledge about ME before attending the program?
        - Internet, media, research, health care services, user organization, other?
- What expectations did you have before attending the program?
- Which topics in the program did you find important/relevant, or less relevant – why was it important /not important?
- Were there any topics you felt were missing? If so, which?
- Have you learned something new from participating in the program and was the program useful – please describe
- Is there anything else you want to share that you think is important about the self-management program

INTERVIEW GUIDE – Focus Group Interview

- How long has it been since you received the diagnosis or your next of kin were diagnosed with ME?
- Please describe your experiences of the self-management support services you have received (or your next of kin)?
    - What was good, and why was it good– please describe
    - What was not good, and why was it not good– please describe
    - Could the self-management support services been different, how, please describe
- Could the self-management support services been further developed, how?
    - The content?
    - The organization?
    - Use of digital tools?
- Is there anything else you want to share about self-management support services, that we haven't discussed? Please tell
